# Conjugation with Methylsulfonylmethane Improves Hyaluronic Acid Anti-Inflammatory Activity in a Hydrogen Peroxide-Exposed Tenocyte Culture In Vitro Model

**DOI:** 10.3390/ijms21217956

**Published:** 2020-10-26

**Authors:** Francesco Oliva, Marialucia Gallorini, Cristina Antonetti Lamorgese Passeri, Clarissa Gissi, Alessia Ricci, Amelia Cataldi, Alessia Colosimo, Anna Concetta Berardi

**Affiliations:** 1 Department of Musculoskeletal Disorders, Faculty of Medicine and Surgery, University of Salerno, 84081 Baronissi, Italy; foliva@unisa.it; 2 Department of Pharmacy, University G. d’Annunzio of Chieti-Pescara, Via dei Vestini 31, 66100 Chieti, Italy; alessia.ricci@unich.it (A.R.); cataldi@unich.it (A.C.); 3 Laboratory of Stem Cells, U.O.C. of Immunohaematology and Transfusion Medicine, Santo Spirito Hospital, 65122 Pescara, Italy; cristinaalp94@gmail.com (C.A.L.P.); gissi.clarissa@spes.uniud.it (C.G.); 4 Faculty of Bioscience and Technology for Food, Agriculture and Environment, University of Teramo, Via R. Balzarini 1, 64100 Teramo, Italy; acolosimo@unite.it

**Keywords:** rotator cuff tears, rotator cuff disease, tendinopathies, inflammation, oxidative stress, hyaluronic acid, methylsulfonylmethane

## Abstract

Rotator cuff tears (RCTs) and rotator cuff disease (RCD) are important causes of disability in middle-aged individuals affected by nontraumatic shoulder dysfunctions. Our previous studies have demonstrated that four different hyaluronic acid preparations (HAPs), including Artrosulfur^®^ hyaluronic acid (HA) (Alfakjn S.r.l., Garlasco, Italy), may exert a protective effect in human RCT-derived tendon cells undergoing oxidative stress damage. Recently, methylsulfonylmethane (MSM) (Barentz, Paderno Dugnano, Italy) has proven to have anti-inflammatory properties and to cause pain relief in patients affected by tendinopathies. This study aims at evaluating three preparations (Artrosulfur^®^ HA, MSM, and Artrosulfur^®^ MSM + HA) in the recovery from hydrogen peroxide-induced oxidative stress damage in human tenocyte. Cell proliferation, Lactate Dehydrogenase (LDH) release, and inducible nitric oxide synthases (iNOS) and prostaglandin E2 (PGE2) modulation were investigated. In parallel, expression of metalloproteinases 2 (MMP2) and 14 (MMP14) and collagen types I and III were also examined. Results demonstrate that Artrosulfur^®^ MSM + HA improves cell escape from oxidative stress by decreasing cytotoxicity and by reducing iNOS and PGE2 secretion. Furthermore, it differentially modulates MMP2 and MMP14 levels and enhances collagen III expression after 24 h, proteins globally related to rapid acceleration of the extracellular matrix (ECM) remodelling and thus tendon healing. By improving the anti-cytotoxic effect of HA, the supplementation of MSM may represent a feasible strategy to ameliorate cuff tendinopathies.

## 1. Introduction

Degenerative nontraumatic rotator cuff tears (RCTs) and rotator cuff disease (RCD) are a common cause of chronic shoulder pain and disability, for which incidence increases with age, starting from the third decade of life, with a maximum epidemiologic peak above the fifth one [1]. Conservative surgery could be a possibility for repairing tendon damages in patients suffering from RCTs and RCD. Among conservative management options, growing evidences indicate that hyaluronic acid (HA) alleviates pain, counteracts the inflammation reaction, and improves function in individuals affected by age-related RCTs [2,3,4,5]. Our group has recently shown that human tendon-derived cells from RCTs promptly respond to different hyaluronic acid preparations (HAPs) in vitro, including Artrosulfur^®^ HA, by enhancing cellular metabolism and by counteracting hydrogen peroxide (H_2_O_2_)-induced cytotoxicity and inflammation [6,7,8]. In detail, it has been shown that Artrosulfur^®^ HA is able to stimulate cell viability and collagen type I synthesis in a dose-dependent manner after 7–14 days of in vitro culture [6]. Furthermore, by modulating the hyaluronan receptor CD44 and the Nrf2 transcription factor-related molecular cascade, this HAP has demonstrated a higher cytoprotective effect in human RCT-derived tendon cells undergoing oxidative stress damage [7,8].

Another product that might have some potential benefit in conservative therapy for RCTs and RCD is methylsulfonylmethane (MSM). This organic compound has shown antioxidant features in human neutrophils, causing a reduced synthesis of prooxidant toxic substances, including hydrogen peroxide [9]. Moreover, MSM has been used for contrasting liver injury in rats and osteoarthritis in humans due to its antioxidant, anti-inflammatory, and antiapoptotic properties [10,11]. Novel data have also suggested that MSM can induce wound healing due to its positive effect on pain relief in patients suffering from tendinopathies [10].

Recent studies have shown that the generation of Nitric Oxide (NO) plays a relevant role in repairing an injured tendon [12]. NO synthesis originates from three distinct forms of nitric oxide synthases (NOSs): the endothelial NOS (eNOS), the neuronal NOS (nNOS), and the inducible NOS (iNOS), with iNOS being the most efficient in NO generation. It has been demonstrated that their enzymatic activity is upregulated in injured tendons and that NO synthesis enhances collagen production during tendon healing [13]. For these reasons, the evaluation of iNOS overexpression, as an indirect marker of increased NO synthesis, is an important parameter to be considered in damaged tendons.

Other studies have suggested that modulation of prostaglandin overproduction could be a potential strategy to treat tendon diseases [14]. As a matter of fact, the lipid mediator prostaglandin E2 (PGE2) seems to be one of the most fundamental in regulating physiological functions and inflammatory responses in the pathogenesis of tendonitis [15], being implicated in all processes leading to cardinal signs of inflammation, such as heat, redness, swelling, and pain [14]. As such, PGE2 represents a valuable marker of inflammation occurrence due to oxidative stress-mediated injury.

The tendons are made up of compact connective tissue formed by a relatively small number of cellular elements that are surrounded by an abundant extracellular matrix (ECM). The most prevalent component of tendon-ECM is collagen type I, alongside smaller proportions of collagen type III. It is well known that, in tendinopathies, a wide disorganization of ECM occurs, including a significant decrease of collagen type I. However, all the molecular mechanisms responsible for the extracellular matrix alteration and collagen type I fibre decreases are still unknown [16] Metalloproteinases (MMPs), such as MMP2 and MMP14, are a family of enzymes required for the normal architecture of ECM and having a pivotal role during tendon injury because they degrade the connective tissue components and modify the ECM [17]. It was supposed that modulation of MMP activity could promote tendon healing [18].

The present in vitro study was designed in order to evaluate the biological effect of three preparations (Artrosulfur^®^ HA, MSM, and their combination-termed Artrosulfur^®^ MSM + HA) in terms of cell recovery from oxidative stress in human RCT-derived tenocyte cultures.

The ability of tendon-derived cells to recover from cytotoxicity induced by H_2_O_2_ treatment was analysed by evaluating different parameters, including cell proliferation, lactate dehydrogenase release, total protein content, and protein expression levels of inducible nitric-oxide synthase (iNOS). To assess the contribution of HAPs in the process of tendon healing, the modulation of metalloproteinases (MMP)-2 and MMP14 as well as of extracellular matrix-related proteins, such as collagen type I and collagen type III, was evaluated. In addition to these parameters, prostaglandin E2 (PGE2) secretion was also evaluated.

## 2. Results

### 2.1. Cell Morphology and Proliferation

**Figure 1:**
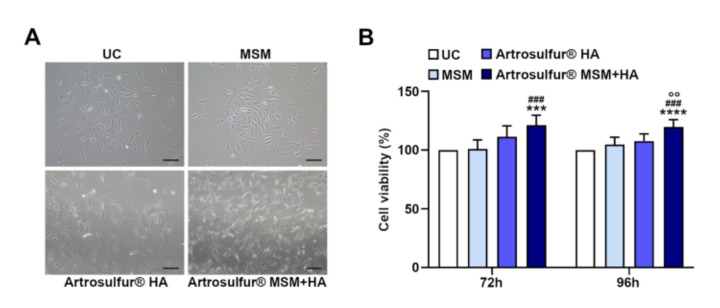
Cell morphology and proliferation (MTT (3-[4,5-dimethyl-thiazol-2-yl-]-2,5-diphenyl tetrazolium bromide) assay) in tendon-derived cells that were treated with methylsulfonylmethane (MSM) (0.3% *w*/*v*), Artrosulfur^®^ hyaluronic acid (HA) (1000 µg/mL), and Artrosulfur^®^ MSM + HA (0.3% *w*/*v* + 1000 µg/mL) for 72 h and 96 h: (**A**) After 96 h of treatment, cell morphology was observed by optical microscopy. Elongated cells are viable and adherent, while rounded floating cells are detached. UC: control untreated cells. Magnification, ×100. (**B**) The graph represents OD (optical density) values in control and treated samples. Data shown are the means (± SD) of three independent experiments. UC: control untreated cells. *** *p* ≤ 0.001 and **** *p* ≤ 0.0001 vs. UC; ^###^ *p* ≤ 0.001 vs. MSM; and °° *p* ≤ 0.01 vs. HA.

At first, we evaluated cell morphology that remained unaltered in all the experimental conditions, with respect to controls (Figure 1A). Then, cell proliferation of the tendon-derived cells was evaluated by MTT (3-[4,5-dimethyl-thiazol-2-yl-]-2,5-diphenyl tetrazolium bromide) assay, following their exposure to MSM alone, Artrosulfur^®^ HA, or Artrosulfur^®^ MSM + HA after 72 and 96 h (Figure 1B). The proliferation rate in cells treated with Artrosulfur^®^ MSM + HA was significantly increased compared either with MSMalone (121% ± 0.8 vs. 101% ± 0.7) (*p* = 0.0003), with Artrosulfur^®^ HA (111% ± 0.9), or with the untreated cells used as a control (100%) (*p* = 0.0002) after 72 h of treatment (Figure 1B). Likewise, following 96 h of treatment, the proliferation rate of cells exposed to Artrosulfur^®^ MSM + HA significantly increased compared either with the untreated cells (119% ± 0.6) (*p* = 0.0001), with MSM alone (104% ± 0.6) (*p* = 0.0004), or with Artrosulfur^®^ HA (107% ± 0.6) (*p* = 0.0037) (Figure 1B).

**Figure 2:**
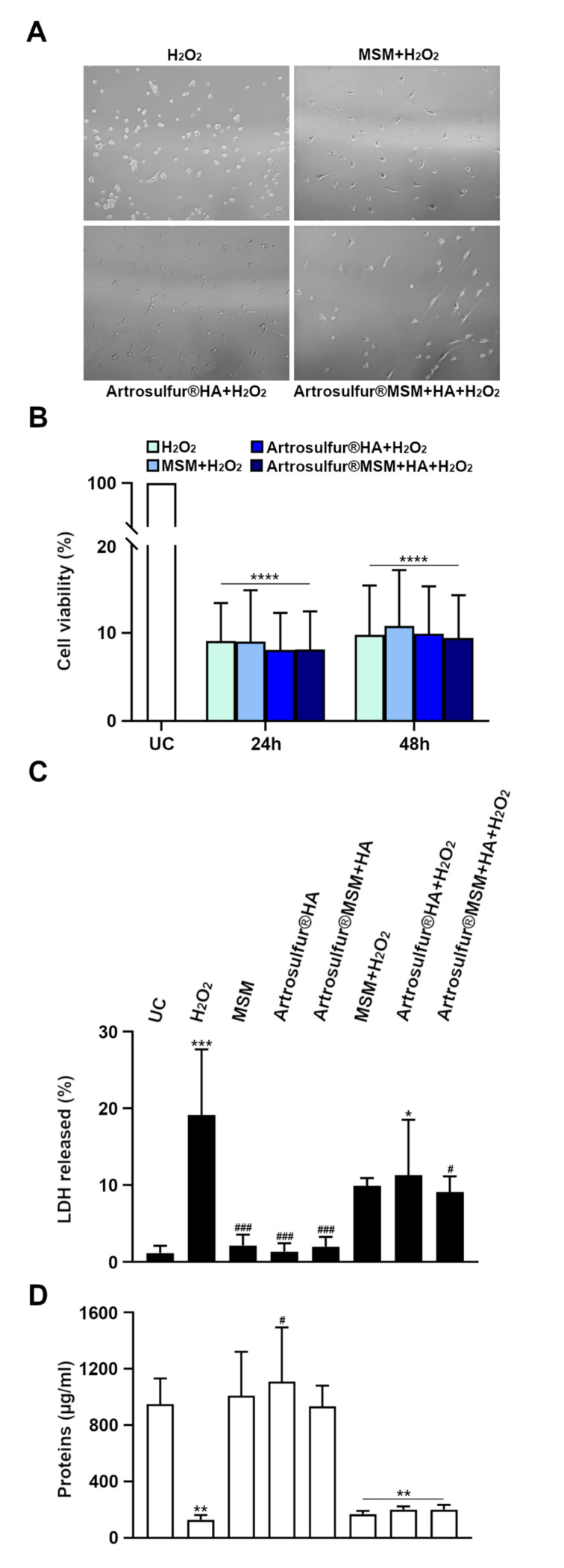
(**A**,**B**) Cell morphology and MTT assay in rotator cuff tear (RCT)-derived tenocytes that were treated with H_2_O_2_ for 0, 24, and 48 h, respectively, with or without MSM, Artrosulfur HA^®^, or Artrosulfur^®^ MSM + HA: (**A**) Cell morphology was observed by optical microscopy after 48 h of treatment. Morphological changes were associated with round-shaped and detached cells (death cell), whereas vital cells were elongated and adhered to the culture plate. (**B**) The graph shows the % of living cells after 0, 24, and 48 h of treatment. (**C**) Amount of Lactate Dehydrogenase (LDH) released from tenocytes in the indicated experimental conditions: bars show the mean percentages ± SD (*n* = 3) of LDH released by tenocytes compared to total LDH released from untreated controls (UC) after 24 h of exposure. (**D**) Total amount of proteins after 24 h of treatment, expressed as µg of proteins/mL of cell lysate. * *p* ≤ 0.05, ** *p* ≤ 0.01, and *** *p* ≤ 0.001, **** *p* ≤ 0.0001 between median values of treated samples and untreated cells after 24 h and 48 h. ^#^ *p* ≤ 0.05 and ^###^ *p* ≤ 0.001, between mean values of treated samples and H_2_O_2_ alone after 24 h.

To investigate the proliferation rate in terms of modulation of cell metabolic activity of tenocytes after exposure to H_2_O_2_ alone or in combination with MSM, Artrosulfur^®^ HA, or Artrosulfur^®^ MSM + HA, the MTT assay was performed. Before running the assay, cell morphological changes after treatments were evaluated under a light phase-contrast microscope (Figure 2A). Generally, the observation revealed that dead round-shaped cells no longer adhering to the culture plate were the most prevalent type in the presence of H_2_O_2_ alone, while vital elongated fibroblast-like cells (similar to untreated controls shown in Figure 1A) prevailed when MSM and, above all, the two HAPs were coadministered together with H_2_O_2_, After 24 h of treatment, cell proliferation was not totally suppressed in response to 2 mM H_2_O_2_ and no substantial change was observed when H_2_O_2_ was administered together with one of the three preparations (Figure 2B). After 48 h of co-treatment, MSM (10.8%), Artrosulfur^®^ HA (9.9%), and Artrosulfur^®^ MSM + HA (9.4%) slightly promoted cell viability, when compared to H_2_O_2_ alone (9.7%).

### 2.2. Lactate Dehydrogenase (LDH) Cytotoxicity Assay and Protein Quantification

We next investigated H_2_O_2_-induced cytotoxicity in each treatment by measuring the level of LDH released and the amount of total protein in the whole cell lysate as an indirect quantification of the cell number increase. The secretion of LDH from RCT-derived tenocytes significantly increased (19%) after H_2_O_2_ treatment with respect to untreated cells (1%) (Figure 2C), thus showing a substantial cytotoxicity. The administration of either MSM alone (9%), Artrosulfur^®^ HA (11%), or Artrosulfur^®^ MSM + HA (9%) counteracted H_2_O_2_-induced cytotoxicity in tenocytes after 24 h, significantly reducing the amount of LDH released (Figure 2C). Among the three preparations, Artrosulfur^®^ MSM + HA seemed to be the most effective in restoring membrane integrity following hydrogen peroxide administration due to the lower amount of lactate produced (*p* = 0.04) (Figure 2C).

In parallel, a sharp decrease of the protein content (93 ± 6 µg/mL) was observed after addition of 2 mM H_2_O_2_ to the tenocyte culture, compared to controls (950 ± 18) (Figure 2D). When the three preparations were coadministered with H_2_O_2_, the levels of proteins content slightly increased (MSM: 168 ± 22 µg/mL, Artrosulfur^®^ HA: 200 ± 22 µg/mL, and Artrosulfur^®^ MSM + HA: 200 ± 33 µg/mL) in the effort to counteract H_2_O_2_-induced cytotoxicity (Figure 2D).

### 2.3. iNOS Expression

**Figure 3:**
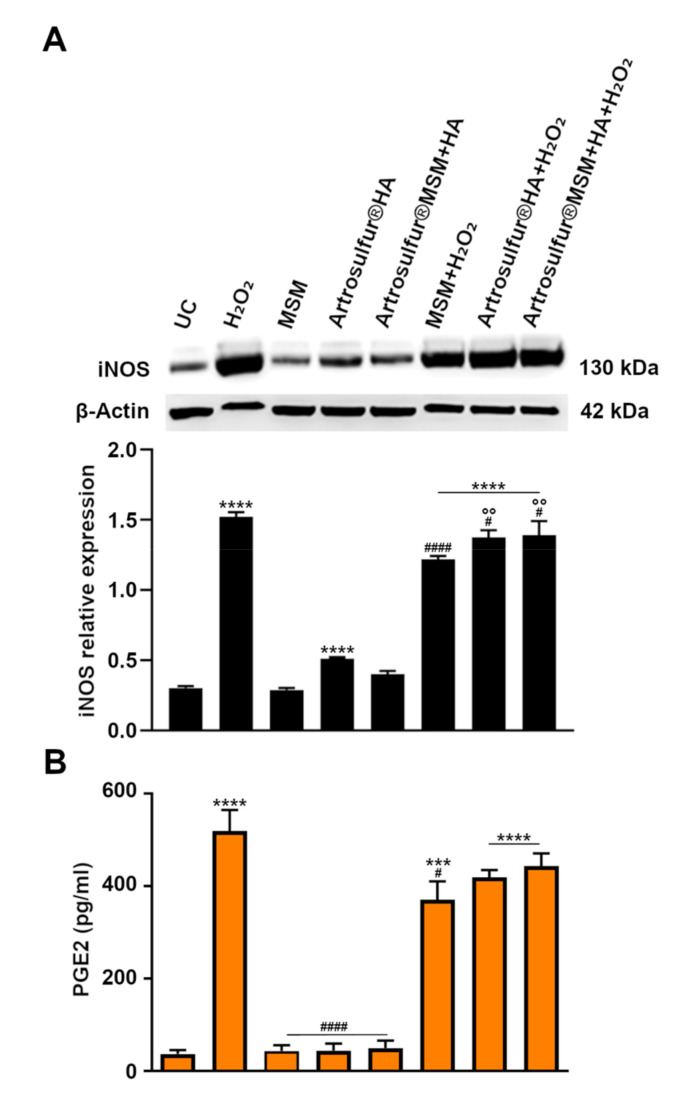
(**A**) Western blot analysis of inducible nitric oxide synthase (iNOS) protein expression in RCT-derived tenocytes in the indicated experimental conditions: β-actin is used as control. Bar graphs display densitometric values expressed as the mean percentage ± SD (*n* = 3) of optical densities of bands normalized on β-actin. *** *p* ≤ 0.001, and **** *p* ≤ 0.0001, between median values of treated samples and untreated cells; ^#^ *p* ≤ 0.05, ^####^ *p* ≤ 0.0001 between median values of HA-treated samples + H_2_O_2_ and H_2_O_2_; and ^°°^ *p* ≤ 0.01, between median values of HA-treated samples + H_2_O_2_ and MSM + H_2_O_2_. (**B**) Prostaglandin E2 (PGE2) secretion from RCT-derived tenocytes in the indicated experimental conditions. Data represent means ± SD. *n* = 3.

The status of H_2_O_2_-induced oxidative stress and cytotoxicity in each treatment was also evaluated by measuring the expression levels of inducible NOS, since it has been shown that antiapoptotic effects of MSM are mediated by its inhibitory effects on iNOS [16]. As shown in Figure 3A, H_2_O_2-_induced oxidative stress dramatically produced elevated levels of the prooxidant enzyme iNOS (1.5-fold increase on β-actin), with respect to untreated cultures (0.3). Conversely, when MSM, Artrosulfur^®^ HA, and Artrosulfur^®^ MSM + HA were administered to H_2_O_2_-stimulated cells, all three formulations significantly decreased iNOS expression compared to H_2_O_2_ alone (1.2-, 1.3-, and 1.3-folds, respectively) (*p* = 0.0001, *p* = 0.04, and *p* = 0.03, respectively).

### 2.4. PGE2 Secretion

PGE2 released from tenocytes was quantified as an evaluation of inflammation occurrence (Figure 3B). In untreated cultures, the amount of PGE2 secreted was assessed at 36 pg/mL, a comparable value with the ones registered when cells were exposed to MSM (43 pg/mL) and the HAP formulations separately (44 pg/mL for Artrosulfur^®^ HA; 49 pg/mL for for Artrosulfur^®^ MSM + HA). As expected, the PGE2 amount dramatically increased (519 pg/mL) when RCT-derived tenocytes were exposed to 2 mM H_2_O_2_. Notably, the presence of MSM alone decreased the cytokine release from H_2_O_2_-exposed cultures up to 370 pg/mL. Likewise, the two HAPs were similarly efficient in decreasing PGE2 amounts (419 pg/mL for Artrosulfur^®^ HA and 443 pg/mL for Artrosulfur^®^ MSM + HA).

### 2.5. Metalloproteinases and Expression of Collagen Proteins

To evaluate the behaviour of fundamental components in tendon tissue and in ECM remodelling, we also quantified the protein expression levels of two metalloproteinases (MMP2 and 14) and of collagen types I and III.

**Figure 4:**
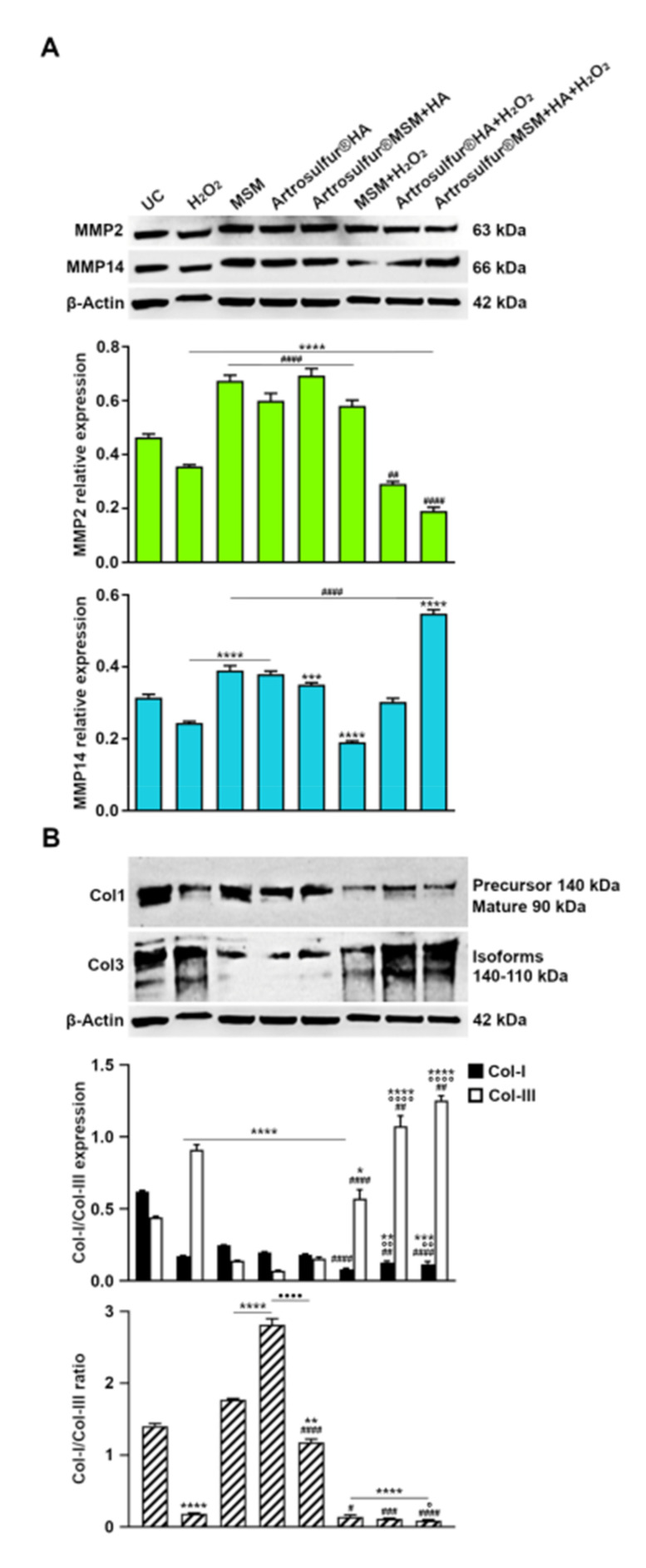
(**A**) Western blot analysis of metalloproteinases 2 (MMP2) and 14 (MMP14) protein expression in RCT-derived tenocytes in the indicated experimental conditions and (**B**) Western blot analysis of collagen type 1 and collagen type III protein expression in RCT-derived tenocytes in the indicated experimental conditions: β-actin is used as control. Bar graphs display densitometric values expressed as the mean percentage ± SD (*n* = 3) of normalized on β-actin. * *p* ≤ 0.05, ** *p* ≤ 0.01, *** *p* ≤ 0.001, and **** *p* ≤ 0.0001, between median values of treated samples and untreated cells; ^#^ *p* ≤ 0.05, ^##^ *p* ≤ 0.01, ^###^ *p* ≤ 0.001, and ^####^ *p* ≤ 0.0001, between median values of HA-treated samples + H_2_O_2_ and H_2_O_2_; ^°^ *p* ≤ 0.05, ^°°^ *p* ≤ 0.01, and ^°°°°^ *p* ≤ 0.001, between median values of HA-treated samples + H_2_O_2_ and MSM + H_2_O_2_.

The H_2_O_2_ exposure lowered the expression of both MMP2 and MMP14 with respect to untreated cultures (Figure 4A). Regarding MMP2 protein, when MSM and HAPs were separately administered to the culture, all formulations significantly enhanced its expression, mainly in the presence of Artrosulfur^®^ MSM + HA. The same trend was observed for MMP14, but to a lesser extent with respect to the untreated control. In the presence of H_2_O_2_ and the three formulations at the same time, the two MMPs had a differential and reversed behaviour. In detail, MMP2 expression levels remained consistently high in the presence of MSM (0.5-fold increase on β-actin), while they significantly decreased compared to H_2_O_2_-treated cells (0.3) when the two HAPs were added to the tenocyte culture (0.2 and 0.1 for Artrosulfur HA^®^ and Artrosulfur^®^ MSM + HA, respectively). Conversely, MMP14 expression amounts were lower than H_2_O_2_-treated tenocytes (0.24) when MSM was added to the culture (0.1), while they increased in the copresence of Artrosulfur HA^®^ (0.3) and mostly of Artrosulfur^®^ MSM + HA (0.5), for which coadministration showed the absolute highest level of expression.

The ratio of collagen type I to collagen type III drastically decreased (0.18) when H_2_O_2_ was added to the tenocyte culture, since an increase in collagen type III expression and a concomitant reduction in collagen type I was observed. Interestingly, the Artrosulfur^®^ MSM + HA formulation better reduced this ratio (0.09-fold increase on β-actin) when compared to MSM (0.14) and Artrosulfur^®^ HA alone (0.11) (Figure 4B).

## 3. Discussion

Despite growing evidence that hyaluronic acid preparations are effective tools in alleviating pain and in counteracting inflammation in individuals suffering from nontraumatic rotator cuff disease, the underlying molecular mechanisms are still poorly understood. Our in vitro findings demonstrate that Artrosulfur^®^ MSM + HA, a combination of the antioxidant compound MSM and HA, may improve the anti-inflammatory and antioxidant cell responses in human RCT-derived tenocyte primary cultures under H_2_O_2_-induced oxidative stress. It should be noted that the HA concentrations used here (1000 µg/mL) were chosen according to our previous study, in which we evaluated Artrosulfur^®^ HA dose-response on tendon-derived cells [6]. Artrosulfur^®^ MSM + HA increased significantly the cell-proliferation rate after 72 h and 96 h of treatment when compared with both Artrosulfur^®^ HA and MSM. These data are concordant with other in vitro studies which showed that HA can induce cell proliferation in other cellular types, such as cartilage tissue. However, in vivo clinical trials showing a proliferative effect of HAPs on tendon-derived cells are still missing [19,20]. In addition, with the exception of our previously published studies regarding Artrosulfur^®^ HA [6,8], the other HAPs were used here for the first time on a human primary tendon-derived cell culture.

Regarding the effect of MSM on tenocytes, previous studies have shown its contribution in stimulating the differentiation of mesenchymal stem cells towards the osteogenic lineage, leading to bone formation [21]. In parallel, Kim and collaborators have showed that MSM exerted an inhibitory effect on cancer cell proliferative activity [22,23]. In the present study, we have demonstrated that MSM in combination with Artrosulfur^®^ HA enhanced the proliferation rate of RCT-derived tenocytes. Consequently, further in vivo studies will be very much worthwhile in order to clarify the role of MSM on cellular proliferation, by itself or in combination with different HA formulations. In our previous study, we showed the ability of Artrosulfur^®^ HA to counteract H_2_O_2_-induced cytotoxicity after just 6 and 24 h of exposure [8]. Accordingly, here, we demonstrate that all three treatments slightly increased cell viability of H_2_O_2_-treated tenocytes over time. Contrarily, in one recent study of Kim and coauthors, MSM administration did not modify cell viability of horse foetal liver cells after H_2_O_2_-induced cytotoxicity [24]. To our knowledge, there are no available data from other studies regarding the recovery of human tenocytes from H_2_O_2_-induced cytotoxicity in terms of increased proliferation rate due to the administration of MSM and HAPs.

In addition, all three treatments significantly reduced the amount of LDH released in the presence of H_2_O_2_, with Artrosulfur^®^ MSM + HA resulting in being the most effective preparation in restoring membrane integrity, after 24 h of H_2_O_2_ exposure. In parallel, the slight increase in protein content that was observed in the presence of all three preparations in concomitance with H_2_O_2_ treatment showed that they are able to contrast H_2_O_2_-induced cytotoxicity, in agreement with our previous findings [8].

Since it has been demonstrated that MSM antiapoptotic effects are mediated by its inhibitory effects on inducible nitric oxide synthase protein and nitric oxide levels [25], expression levels of the prooxidant enzyme iNOS were determined. While H_2_O_2_-mediated oxidative stress produced significant increased levels of iNOS, the administration of MSM, Artrosulfur^®^ HA, and Artrosulfur^®^ MSM + HA significantly decreased iNOS amount, even if normal levels were not restored. Considering the antioxidant function of MSM and HA, we further demonstrated that the redox balance was improved by all three preparations. To note, when MSM was administered in parallel with H_2_O_2_, a significant decrease in iNOS levels was observed if compared to H_2_O_2_-induced cytotoxicity. This decline in iNOS expression was not so noticeable when MSM was administered together with HA. Thus, although with different efficiencies, MSM and all HAPs may alleviate oxidative stress in primary tenocyte cultures. This result is particularly intriguing since it has been demonstrated that nitric oxide (NO) favours tendon healing at the molecular level by increasing collagen synthesis after 7 days of treatment in an in vitro tendon cell culture [26]. As it is well-known, NO expression is in turn enhanced by the high levels of nitric oxide synthases (NOS) [27]. Furthermore, iNOS seems to play a fundamental role in tendinopathies, since its overexpression in human tenocytes has been broadly reported to stimulate transcription and translation of a number of extracellular matrix-related genes, such as collagens I, II, and IV; biglycan; decorin; laminin; and MMP-10 [27]. However, to offer deeper insight into NO effects in tendinopathies, further studies are necessary.

Our additional results showed a significant increased production of PGE2 in response to H_2_O_2_ when compared to the untreated control, thus confirming a cytotoxic effect of the treatment. Prostaglandin is one of the eicosanoids involved in many chronic inflammatory diseases, including tendinopathies [28]. The levels of PGE2 in cells treated with Artrosulfur^®^ HA and Artrosulfur^®^ MSM + HA, even if not in a statistically significant manner, decreased as compared to tenocytes exposed to H_2_O_2_ alone. To note, MSM induced a significant decrease of PGE2 compared with H_2_O_2_ alone in agreement with a previous study, thus confirming the anti-inflammatory effects of MSM [29]. Furthermore, it is intriguing that the combination of Artrosulfur^®^ MSM + HA did not improve the reduction of PGE2 expression compared to MSM alone, that resulted in the most efficient treatment in reducing cytotoxicity due to H_2_O_2_ administration. Interestingly, the synthesis of both iNOS and PGE2 increased or decreased simultaneously in the presence of all three preparations, showing a similar trend. As such, PGE2 and iNOS might be coproduced during H_2_O_2_-mediated oxidative stress. Although the administration of MSM seemed to be the most effective, all treatments, including Artrosulfur^®^ HA and Artrosulfur^®^ MSM + HA, were found to be able to reduce inflammatory biomarkers. In this experimental setting, the combination of MSM with HA (i.e., Artrosulfur^®^ MSM + HA) did not provide better improvement in alleviating the inflammatory process in tenocytes with respect to the single MSM administration. Increased levels of PGE2 and NO have also been shown in the early cell response towards tendon injury. However, persistent and unsolved inflammation with sustained excessive production of mediators, growth factors, and enzyme degradation occurrence could result in further damage and aberrant fibrotic repair [30]. An imbalance in the levels of inflammatory mediators and proteolytic enzymes could lead to progressive tendon ECM deterioration, impaired healing, and rupture. In the present study, we show that hyaluronic acid preparations are highly active in reducing the inflammatory biomarkers associated to oxidative stress. Because the overproduction of pro-inflammatory factors can be harmful, pharmacological interference with the NO and PGE2 cascade represents a promising strategy for therapeutic intervention in inflammatory disorders., Also, the obtained results have shown for the first time that MSM, Artrosulfur^®^ HA, and Artrosulfur^®^ MSM + HA attenuate the overproduction of iNOS and PGE2 on RCT-derived tendon cells, suggesting that all three formulations may mitigate deleterious inflammation, without ablating PGE2 and iNOS syntheses that are needed for physiological tendon healing and repair.

Given the key role of ECM components in tendon tissue, we also investigated matrix metalloproteinase expression levels, such as MMP2 and MMP14, and collagen type I and collagen type III proteins, broadly known as key components and mediators of ECM remodelling. As a matter of fact, MMP2 is involved in the degradation and remodelling of specific forms of collagen in wound healing [31]. In detail, an enhanced MMP2 expression and a decreased collagen production in tenocytes suggest an altered process of tendon healing. In this study, MSM administration in H_2_O_2_-treated cells enhanced MMP2 expression, while Artrosulfur^®^ HA and Artrosulfur^®^ MSM + HA had an inhibitory effect. Interestingly, Artrosulfur^®^ MSM + HA seems to induce a higher inhibition of MMP2 levels. While it is known that MMP14 cleaves especially collagen I type, more recently, it has been demonstrated that MMP14 is capable of cleaving additional macromolecules for releasing collagen fibrils from fibripositors and for promoting the formation of new collagen fibre [32]. As such, MMP14 is required for proper tendon development. In addition, MMP14 is involved in several other cell processes, such as migration and proliferation [33]. In this study, MMP14 expression was found to be decreased following H_2_O_2_-mediated oxidative stress while the coadministration of MSM induced a deeper decrease in the level of protein expression. On the contrary, Artrosulfur^®^ HA and Artrosulfur^®^ MSM + HA administration counteracts the H_2_O_2_-mediated damage by inducing a significant increase of MMP14 expression level. Interestingly, Artrosulfur^®^ MSM + HA induced a higher level of expression of MMP14. These results are concordant with previous studies that showed that MMP2 an MMP14 act in concert and contribute to collagen turnover [34].

Regarding the normal expression of the two other investigated extracellular matrix-related proteins, collagen type I and type III, the first one is the most abundant form in healthy tendons while collagen type III is the first that is synthesized during the tendon healing process. In the presence of H_2_O_2_-induced cytotoxicity, the ratio of collagen type I to collagen type III significantly decreased. In a previous study, it was demonstrated that, during oxidative stress, in supraspinatus tendon and humeral head of Sod1^–/–^ male mice, the expression of collagen type I significantly decreased without any significant change in collagen type III expression [35]. Notably, the Artrosulfur^®^ MSM + HA preparation better reduced this ratio compared to Artrosulfur^®^ HA. MSM induced a decreased expression of both collagen type I and type III with a consequent decrease in their ratio if compared to the solely H_2_O_2_ treatment. This ratio was slightly higher than the one observed when the other two HAPs (Artrosulfur^®^ HA and Artrosulfur^®^ MSM + HA) were administered.

Following tendon injury, collagen type III is the first form that is synthesized at the wound site in the initial phase of healing, while it is replaced by a large deposition of collagen type I during the following stages of tendon recovery (remodelling and tissue maturation) [36]. Our result suggests that, just after one day, all three preparations accelerated the second phase of healing process of an injured tendon, i.e., the reparative (proliferation) phase, characterized by cellularity and matrix production, mostly formed by collagen type III as described [36]. Moreover, these data are concordant with our previous study that demonstrated a consistent increase of collagen type I, after only seven days of HAPs administration [6].

One limitation of the present study is that the combination of MSM with Artrosulfur^®^ HA does not necessarily indicate their interplay. It would be interesting to test different doses of each compound, when used in combination, to evaluate their effect in term of toxicity, cell proliferation, and anti-inflammatory activity on tenocyte cultures. Thus, the present preliminary study could be pivotal for laying the grounds about the possibility of their effectiveness in tendinopathies.

## 4. Materials and Methods

### 4.1. Cell Culture

Human tenocytes derived from rotator cuff tendons, isolated from the same 10 patients described in our previous work [14], and cryopreserved in vials in liquid nitrogen were used. The cells at passage 0 were thawed out and promptly cultured to avoid phenotype changes caused by later passages [6,8,37,38].

### 4.2. Cell Treatment

Human tenocytes were spread in 96-well (0.5 × 10^4^ cells/well) and 6-well (0.5 × 10^5^ cells/well) tissue culture plates (Falcon^®^, Corning Incorporated, Brooklyn, NY, USA) and were left to adhere overnight. To evaluate the effect of HAPs on cell morphology and proliferation (for the MTT assay, see below), cells were cultured in 96-well plates and exposed to MSM alone or to the HAP formulations separately, namely Artrosulfur^®^ HA (M.W. 1200 KDa) or Artrosulfur^®^ MSM + HA (M.W. 1200 KDa) (Table 1). The final concentration of the HAPs was 1000 µg/mL in complete α-MEM, supplemented with 10% FBS (Fetal Bovine Serum) and 1% penicillin/streptomycin (Gibco, Invitrogen, Life Technologies, CA, USA), and the pH was adjusted to 7, as already reported [6,8]. To evaluate the effect of the MSM compound by itself in comparison with the other two HAPs, the same MSM concentration in the formulation of 1000 μM Artrosulfur^®^ MSM + HA was used in the assay (final concentration 0.3% *w*/*v*). Untreated cells were used as control.

### 4.3. H_2_O_2_ Treatment

Hydrogen peroxide, 2 mM, was used to induce cytotoxicity as previously described [8]. After being seeded for 24 h in 6-well plates at a cell density of 2 × 10^4^ cells/well in complete α-MEM, the medium was discarded and the cultured cells were treated with H_2_O_2_ with or without MSM, Artrosulfur HA^®^, or Artrosulfur^®^ MSM + HA up to 24 h. A negative control was also investigated by incubating cells in the absence of both H_2_O_2_ and the HAPs (untreated cultures, UC).

### 4.4. MTT Assay for cell Proliferation

The proliferation assay was performed in 96-well plates (5 × 10^3^ cells/well) after 72 and 96 h. Tenocytes were cultured in complete α-MEM medium and exposed to MSM, Artrosulfur HA^®^, or Artrosulfur^®^ MSM + HA. In further experiments, cells were subsequently incubated with 2 mM H_2_O_2_ for 0, 24, and 48 h, with or without MSM, Artrosulfur HA^®^, or Artrosulfur^®^ MSM + HA. At the established experimental times, the medium was discarded and replaced with a fresh one containing 0.5 mg/mL MTT (3-[4,5-dimethyl-thiazol-2-yl-]-2,5-diphenyl tetrazolium bromide) (Sigma-Aldrich, MO, USA) and cells were incubated as previously described [39]. The absorbance was measured at 540 nm using a Multiscan GO microplate spectrophotometer (Thermo Fisher Scientific, MA, USA). The percentage of metabolically active cells was calculated as already reported [39].

### 4.5. Lactate Dehydrogenase (LDH) Release Assay

After 24 h of culture in presence of H_2_O_2_ and eventual HAPs, cell supernatants were analysed by the CytoTox 96^®^ Non-Radioactive Assay (Promega Corporation, Fitchburg, WI, USA) to quantify cytotoxicity occurrence. The assay measures the amount of LDH released following cell lysis using a 30-min coupled enzymatic assay. The LDH release was analysed as previously reported [8].

### 4.6. Cell Lysis and Protein Extraction

After having treated cells in 25-cm^2^ flasks for 24 h, cells were trypsinized, centrifuged, and collected in cold PBS (phosphate buffer). Cell pellets were lysed, and the proteins were extract as previously described [8]. A bicinchoninic acid assay (QuantiPro™ BCA Assay kit, Sigma-Aldrich, St. Louis, MO, USA) was used to determine protein concentration.

### 4.7. Prostaglandin E2 Secretion

After 24 h, cell supernatants were collected from the 96-well plates used for the proliferation assay (MTT) and analysed for PGE2 secretion. A commercial ELISA kit (Enzo Life Sciences, Farmingdale, NY, USA) was used to determine the amount (pg/mL) of PGE2 in the culture media, according to the manufacturer’s instructions. A Multiscan GO microplate spectrophotometer (Thermo Fisher Scientific, MA, USA) was then used to determine the optical density values (absorbance at 405 nm). The PGE2 concentration was determined in each sample by comparing those values to a standard curve using the Prism5 software (GraphPad, San Diego, CA, USA). The obtained values were normalized on the O.D. (optical density) values obtained from the MTT assay and are expressed as means of absolute amounts of PGE2 secreted/well (ng).

### 4.8. Western Blot Analysis

Tenocytes were lysates, and 15 µg of each sample was separated on a 4–20% SDS-PAGE Gel by electrophoresis (ExpressPlus™ 10x8, GenScript Biotech Corporation, Nanjing, China). After running, the samples were transferred to nylon membranes, as already described [8]. The membranes were incubated in the presence of mouse monoclonal anti-β-actin (1:10,000) (Sigma-Aldrich, St. Louis, MO, USA), mouse monoclonal anti-collagen 1A1 (1:200), collagen 3A1 (1:100), mouse monoclonal anti-iNOS (1:200), mouse monoclonal anti-MMP2 (1:200) (all from Santa Cruz Biotechnology, Santa Cruz, CA, USA), and rabbit monoclonal anti-MMP14 (1:1000) (purchased from Abcam). After an overnight incubation at 4 °C with primary antibodies under gentle shaking, membranes were then probed with specific IgG horseradish peroxidase (HRP)-conjugated secondary antibodies and bands were identified by chemiluminescence as previously described [8]. At least three independent experiments were performed for each protein. Results are expressed as mean values ± S.D. of normalized densitometric values on β-actin.

### 4.9. Statistical Analysis

Statistics were performed using one-way analysis of variance (ANOVA) followed by Tukey’s multiple comparison test by means of the Prism 5.0 software (GraphPad, San Diego, CA, USA). The results are the mean values ± SD. Values of *p* ≤ 0.05 were considered statistically significant.

## 5. Conclusions

Overall, the presented experimental results showed, for the first time, an induction of ECM remodelling by all the HAPs and MSM alone, highlighting Artrosulfur^®^ MSM + HA as the best preparation at counteracting H_2_O_2_-induced cytotoxicity and accelerating matrix remodelling. Clearly, further in vitro studies are required to investigate the molecular mechanisms linking MSM and HA in targeting key molecular effectors. As well, additional in vivo studies are crucial to prove that the combination of HA with the antioxidant MSM may improve clinical outcomes of rotator cuff tendinopathies in terms of anti-inflammatory and antioxidant functions.

Growing clinical evidences suggest that HA may induce a prompt relief in different painful human tendinopathies. In a recent prospective multicentre clinical trial, it has been proved that three ultrasound-guided HA injections ameliorated tendon architecture and neovascularization as well as sagittal tendon thickness in patients suffering from both Achilles and patellar tendinopathies. These improvements in tendon features are linked to the role of HA in promoting a disease-modifying effect [40]. Currently, Artrosulfur^®^ HA is the only preparation available on the market, even though there are no published data on its use in the clinic. In this respect, our research opens the way to prospective human clinical trials enrolling patients suffering from several forms of tendinopathies.

## Figures and Tables

**Table 1:** Features of hyaluronic acid preparations tested and MSM.

Commercial Name	Active Substance	Molecular Weight	Source	Doses Tested	Manufacturer
Artrosulfur^®^ HA	Linear Sodium Hyaluronate	1200 KDa	Bacterial Fermentation	1000 ug/mL	Alfakjn S.r.l., Garlasco (PV) Italy
MSM	Methylsulfonylmethane	/	Oxidation of DMSO with hydrogen (H_2_O_2_) and purified by distillation	3125 ug/mL	Barentz, Paderno Dugnano (MI), Italy
Artrosulfur^®^ HA + HA	Linear Sodium Hyaluronate + Methylsulfonylmethane (5% *w*/*v*)	1200 KDa	Bacterial Fermentation	1000 ug/mL + 3125 ug/mL	Alfakjn S.r.l., Garlasco (PV) Italy
